# Characterization of vB_ApiM_fHyAci03, a novel lytic bacteriophage that infects clinical *Acinetobacter* strains

**DOI:** 10.1007/s00705-019-04284-z

**Published:** 2019-05-23

**Authors:** Elsi Pulkkinen, Anu Wicklund, Joseph M. O. Oduor, Mikael Skurnik, Saija Kiljunen

**Affiliations:** 1 Division of Clinical Microbiology, HUSLAB, University of Helsinki and Helsinki University Hospital, Helsinki, Finland; 2 Human Microbiome Research Program, Faculty of Medicine, University of Helsinki, Helsinki, Finland; 3 Department of Medical Microbiology, KAVI-ICR, University of Nairobi, Nairobi, Kenya

## Abstract

We present here the isolation and characterization of *Acinetobacter pittii* phage vB_ApiM_fHyAci03 (fHyAci03), which belongs to the family *Myoviridae*. The fHyAci03 genome was found to be 165,975 bp in length and predicted to contain 255 genes. While the whole genome was 92.4% identical to *Acinetobacter baumannii* phage KARL-1, phylogenetic analysis based on phage long distal tail fiber amino acid sequences assigned fHyAci03 and KARL-1 to different subclusters, reflecting their different host species. Together with phylogenetic analysis, genome comparisons indicated that fHyAci03 is a novel member of the subfamily *Tevenvirinae*. Host range experiments revealed that fHyAci03 could infect two clinical strains of *Acinetobacter nosocomialis* and six clinical strains of *A. pittii.* Thus, fHyAci03 is a novel lytic phage that infects clinical *Acinetobacter* strains and represents a potential new candidate to be used in phage therapy.

**Electronic supplementary material:**

The online version of this article (10.1007/s00705-019-04284-z) contains supplementary material, which is available to authorized users.

The genus *Acinetobacter* includes multiple nosocomial opportunistic pathogens. Within this genus, *A. baumannii*, *A. pittii*, and *A. nosocomialis* are the most frequently isolated species from hospitalized patients around the world [1]. Recently, these bacteria have become a public health concern because of the growing tendency to develop antibiotic resistance [2]. Due to the continued increase in multidrug-resistant (MDR) bacterial strains, there has been a great deal of recent interest in phage therapy research.

fHyAci03 was isolated from a municipal sewage sample collected in Hyvinkää, Finland, using clinical *A. pittii* strain #5565 (obtained from HUSLAB, Helsinki, Finland) as the host. The morphology of fHyAci03 was examined by transmission electron microscopy as described previously [3] (Supplementary Fig. 1). The dimensions of the prolate head were 111 ± 5.2 nm (length) and 82 ± 5.1 nm (width), and the tail length was 90 ± 4 nm. Dimensions were calculated based on ten virions. Together with the genomic information, the morphological characteristics indicated that fHyAci03 belongs to the family *Myoviridae* and the subfamily *Tevenvirinae*. fHyAci03 host range was tested with 48 clinical *Acinetobacter* strains (Supplementary Table 1). Host range experiments revealed that fHyAci03 could infect two out of three *A. nosocomialis* strains, and six out of 18 *A. pittii* strains that were tested. Phage DNA was isolated using an Invisorb Spin Virus DNA Minikit (Stratec Biomedical). Next-generation sequencing was performed at the Institute for Molecular Medicine Finland (FIMM), using a DNA library that was constructed using a Nextera Sample Prep Kit (Illumina, San Diego, CA, USA). Paired-end sequencing was done using an Illumina MiSeq sequencer (Illumina, San Diego, CA, USA) with a read length of 300 nucleotides. Draft sequences were assembled using two pipelines in parallel, Geneious software, version 10.1, and the A5-miseq integrated pipeline [4]. The average whole-genome read coverage was 42.9x. Results from both pipelines were compared to identify a single consensus sequence. To manually verify the fidelity of the assembly, the reads were mapped back to the contigs using Geneious (mean coverage 43.0x, range, 11-96x). The genome was found to be 165,975 bp in length, with a mean GC content of 36.8%. The genome was annotated using Geneious software, RAST [5], BLASTP [6], ARAGORN [7], tRNAscan-SE version 2.0 [8], ResFinder-3.1 [9], and VirulenceFinder-2.0 [10]. fHyAci03 contained 255 predicted genes, eight of which code for tRNAs (Supplementary table S2) and 247 for proteins (Fig. 1). No toxin-, virulence-factor-, antibiotic-resistance-, or lysogeny-related genes were found, indicating that fHyAci03 is strictly lytic and a potential new candidate for phage therapy. A BLASTn search revealed that the most closely related phages belonged to the subfamily *Tevenvirinae* (Supplementary Table S3). Alignment with EMBOSS Stretcher showed that fHyAci03 is 92.4% identical to *A. baumannii* phage KARL-1 (MH713599.1) [11]. Prior to the sequence comparisons, the sequence of KARL-1 was rearranged with respect to its orientation and starting point to align maximally with the T-even phages. Genome-wide nucleotide phylogenetic analysis using VICTOR (Virus Classification and Tree Building Online Resource) [12] placed fHyAci03 in the same subcluster with KARL-1 (Fig. 2A). Further phylogenetic analysis based on phage tail fiber amino acid sequences using VICTOR assigned fHyAci03 and KARL-1 to different subclusters (Fig. 2B), reflecting the difference in host species. Comparisons of conserved gene product content between fHyAci03, KARL1, and other members of the subfamily *Tevenvirinae*, were performed using CoreGenes3.5 [13] (Supplementary Table S4). Based on shared gene content (96.76 % shared homologs) and nucleotide sequence similarity, we suggest that fHyAci03 and KARL-1 could comprise a new genus within the subfamily *Tevenvirinae*, for which we propose the name “*FHyAci03virus*” after the first sequenced isolate. To conclude, fHyAci03 is a novel lytic phage that, with further characterization, could represent an interesting new candidate for phage therapy.

**Fig. 1:**
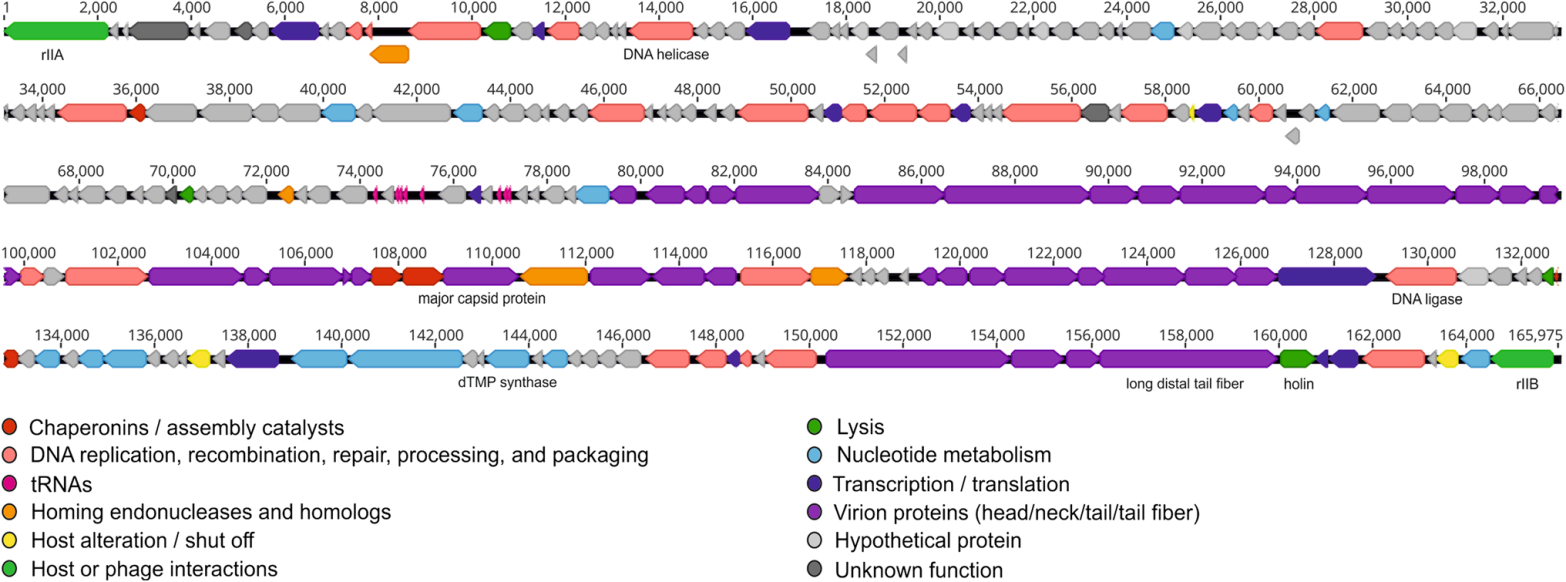
Annotated genome map of fHyAci03. The predicted 247 protein-coding and eight tRNA genes are colour coded according to the key. Some examples of genes that are conserved within the subfamily *Tevenvirinae* are indicated

**Fig. 2:**
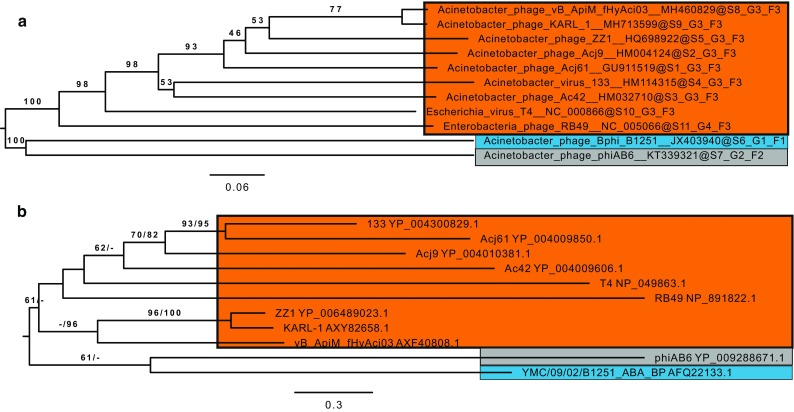
Phylogenetic trees based on the whole-genome nucleotide sequences (a) and amino acid sequences of the tail fiber proteins (b) showing the relationships within the subfamily *Tevenvirinae* and between the families *Myoviridae*, *Podoviridae*, and *Siphoviridae*. Phages are color coded according to family/subfamily. Orange, *Myoviridae*, *Tevenvirinae*; blue, *Siphoviridae*; grey, *Podoviridae*

**Nucleotide sequence accession number**

The genomic sequence of fHyAci03 has been deposited in the GenBank database under the accession number MH460829.1.

## Electronic supplementary material

Below is the link to the electronic supplementary material.

Supplementary material 1 (PDF 643 kb)
