# Supplementary material for: Characterization of vB_ApiM_fHyAci03, a novel lytic bacteriophage that infects clinical Acinetobacter strains
Source: Arch Virol. 2019 May 23;164(8):2197–9. doi: 10.1007/s00705-019-04284-z (PMC6591195; doi:10.1007/s00705-019-04284-z)
Supplement: Supplementary file 1 — Supplementary material 1 (PDF 643 kb) [file 705_2019_4284_MOESM1_ESM.pdf]

Characterization of fHyAci03, a novel lytic bacteriophage that infects clinical *Acinetobacter* strains

Archives of Virology

Elsi Pulkkinen[1,2], Anu Wicklund[1,2], Joseph M. O. Oduor[2,3], Mikael Skurnik[1,2], and Saija Kiljunen[2]

[1]Division of Clinical Microbiology, HUSLAB, University of Helsinki and Helsinki University Hospital, Helsinki, Finland.

[2]Department of Bacteriology and Immunology, Medicum, Research Programs Unit, Immunobiology Research Program, University of Helsinki, Helsinki, Finland

[3]Department of Medical Microbiology, KAVI-ICR, University of Nairobi, Nairobi, Kenya

Corresponding author:

Saija Kiljunen, saija.kiljunen@helsinki.fi

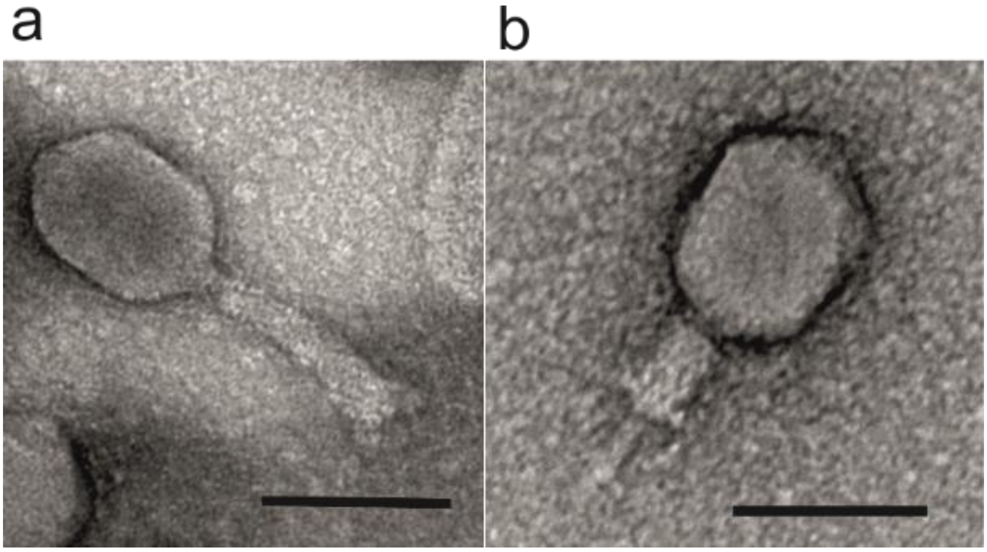

Supplementary Fig. 1 Electron micrographs of negatively stained vB_ApiM_fHyAci03 (fHyAci03) particles. Phage particles with non-contracted (a) and contracted (b) tails are shown. Samples were examined at the Electron Microscopy Unit (Institute of Biotechnology, University of Helsinki, Helsinki, Finland) with a JEOL JEM-1400 transmission electron microscope JEOL Ltd., Tokyo, Japan. Bars represent 100 nm

Supplementary Table 1 Host range of fHyAci03

***Acinetobacter* species (*n*)**	**fHyAci03 sensitivity**	
	**Sensitive**	**Resistant**
A. baumannii (17)	0	17
A. calcoaceticus (1)	0	1
A. junii (1)	0	1
A. lwoffii (3)	0	3
A. nosocomialis (3)	2	1
A. pittii (18)	6	12
A. radioresistens (2)	0	2
A. ursingii (3)	0	3

Sensitive (EOP>10^{-3}), resistant (EOP<10^{-6}). An EOP of 1 was equivalent to 5.3×10^{8} PFU/ml with *Acinetobacter pittii* strain #5565 as the host

Supplementary Table S2 tRNAs, their positions in fHyAci03 genome, products, and anticodons

tRNA #	Start position	End position	Amino acid	Anticodon
1	77217	77148	Met	CAT
2	77140	77065	Cys	GCA
3	76985	76908	Leu	TAG
4	75353	75283	Thr	TGT
5	74994	74904	Ser	TGA
6	74898	74822	Pro	TGG
7	74812	74737	Phe	GAA
8	74354	74280	Trp	CCA

Supplementary Table S3 Whole genome BLASTn comparison with related *Acinetobacter* phages

Related *Acinetobacter* phages	#genes	Query coverage (%)	Identity-%	Accession-no
KARL-1	253	94	98	MH713599.1
ZZ1	256	55	75	HQ698922.4
Acj9	253	52	71	HM004124.1
Acj61	241	46	72	GU911519.1
Ac42	255	29	69	HM032710.1
133	257	18	74	HM114315.1

Supplementary Table S4 Comparisons of conserved gene product content using CoreGenes3.5

		fHyAci03	
**Accession-no**	**Name**	**#homologs**	**%in common**
MH713599.1	KARL-1	239	96.76
HQ698922.4	ZZ1	190	76.92
HM004124.1	Acj9	192	77.73
GU911519.1	Acj61	178	72.06
HM032710.1	Ac42	154	62.35
HM114315.1	133	152	61.54
NC_000866.4	T4	113	45.75
NC_005066.1	RB49	99	40.08
